# An Overview of Structural Characteristics in Problematic Video Game Playing

**DOI:** 10.1007/s40429-017-0162-y

**Published:** 2017-07-13

**Authors:** Mark D. Griffiths, Filip Nuyens

**Affiliations:** International Gaming Research Unit, Psychology Department, Nottingham Trent University, 50 Shakespeare Street, Nottingham, NG1 4FQ UK

**Keywords:** Gaming addiction, Problematic gaming, Structural characteristics, Internet gaming disorder, Gambling addiction

## Abstract

**Purpose of Review:**

There are many different factors involved in how and why people develop problems with video game playing. One such set of factors concerns the structural characteristics of video games (i.e., the structure, elements, and components of the video games themselves). Much of the research examining the structural characteristics of video games was initially based on research and theorizing from the gambling studies field. The present review briefly overviews the key papers in the field to date.

**Recent Findings:**

The paper examines a number of areas including (i) similarities in structural characteristics of gambling and video gaming, (ii) structural characteristics in video games, (iii) narrative and flow in video games, (iv) structural characteristic taxonomies for video games, and (v) video game structural characteristics and game design ethics. Many of the studies carried out to date are small-scale, and comprise self-selected convenience samples (typically using self-report surveys or non-ecologically valid laboratory experiments).

**Summary:**

Based on the small amount of empirical data, it appears that structural features that take a long time to achieve in-game are the ones most associated with problematic video game play (e.g., earning experience points, managing in-game resources, mastering the video game, getting 100% in-game). The study of video games from a structural characteristic perspective is of benefit to many different stakeholders including academic researchers, video game players, and video game designers, as well as those interested in prevention and policymaking by making the games more socially responsible. It is important that researchers understand and recognize the psycho-social effects and impacts that the structural characteristics of video games can have on players, both positive and negative.

## Introduction

Problematic video game playing is multifaceted rather than a unitary phenomenon. Consequently, many factors may come into play in various ways and at different levels of analysis (e.g., psychological, biological, social, situational, structural). Put very simply, there are many different factors involved in how and why people develop problems with video game playing. Central to this view is that no single level of analysis is considered sufficient to explain either the etiology or the maintenance of video game playing behavior. This perspective asserts that all research is context-bound and should be analyzed from a biopsychosocial perspective [1]. One set of factors that are arguably central to understanding video game playing behavior is that of the structure, components, and elements of the video games themselves (i.e., structural characteristics). Over the last decade or so, a small body of both theoretical and empirical papers has examined the role that structural characteristics play in the acquisition, development, and maintenance of video game playing and problematic video game playing. The present paper briefly overviews the key papers in the field to date.

## Similarities in Structural Characteristics of Gambling and Video Gaming

Much of the theoretical and empirical work on structural characteristics in video games has borrowed concepts and terminology from the gambling studies field. Work in the 1990s by Griffiths [2, 3] demonstrated that gambling activities vary considerably in terms of their structural characteristics, such as the probability of winning, the length of the interval between the wagering on an activity and the result of the wager (i.e., event frequency), the number of wagers that can be made at any one time, the size of stake, the size of the jackpot, and the use of the near wins. Structural variations have also been observed for specific forms of gambling such as slot machines where there are structural differences in terms of color, sound effects, and theme which can influence the attractiveness of the games being played [4, 5, 6•]. Applied to the playing of video games, structural features may have implications for gamers’ motivations and the potential “addictiveness” of gaming activities [7].

A number of authors have noted the structural, behavioral, and psychological similarities between specific forms of gambling games (i.e., slot machines) and video games [8–10, 11•, 12]. For instance, Fisher and Griffiths [9] asserted playing slot machines and video games comprise many similarities both structurally and behaviorally including (i) players having to respond to visual stimuli that are controlled by a software loop, (ii) the need for players to have good hand-eye coordination and concentration to play the game, (iii) the rapid speed of the games which to some extent require skillful play (but much more pronounced in video game play), (iv) players being provided with visual and aural rewards when winning, (v) the accumulation of more points/money when winning, and (vi) the potential attention and approval by the player’s peer group when playing.

In the gambling studies field, there are now dozens of published experiments that have manipulated various structural characteristics and demonstrated that such features can prolong gambling compared to games without such features. This includes many experiments demonstrating that “near misses” can increase arousal levels and facilitate repeated gambling [13–26], as well as experiments showing persistent gambling can be influenced by such structural characteristics as jackpot size [18], big wins [22], small wins [27], stake size [28], music [29, 30], and color [30]. Karlsen [11•] compared the psycho-structural elements that contribute to excessive gambling and video gaming, focusing particularly on the elements of the near miss and entrapment. In qualitative interviews with 12 *World of Warcraft* (*WoW*) players (described as “heavy users”), Karlsen reported that near misses and entrapment were present in *WoW* (although not as strong as found in gambling) and influenced by other elements such as social engagement and competition (elements that might have a strong effect on propensity to play excessively).

## Structural Characteristics in Video Games

Griffiths and Wood [31] in a review of gambling and gaming noted that key structural characteristics are the in-built rewards that have the potential to induce repetitive behavior via the partial reinforcement effect [32]. This is arguably one of the most important psycho-structural mechanisms of playing video games and slot machines in that the rewards (i.e., reinforcements) are intermittent (i.e., individuals keep playing with the hope that the next reward is “just around the corner” [31]). When designing attractive and appealing video games, knowledge concerning the partial reinforcement effect gives video game designers a mechanism to facilitate persistent play. As Griffiths and Wood [31] argue, the reinforcement magnitude is important and the bigger the in-game rewards, the faster the gamer is likely to play, and a greater resistance to extinction will be experienced. There are many different reinforcements within gaming including intrinsic rewards (e.g., mastering the game, attempting to better one’s own highest score in-game, bettering the scores of others, getting one’s name in the “hall of fame”) and extrinsic rewards (e.g., receiving admiration from one’s peers for good playing), and it is likely that different structural characteristics are differently rewarding for different gamers.

The first paper to empirically examine multiple structural characteristics in video game playing was by Wood, Griffiths, Chappell and Davies [33•]. In a study comprising 382 gamers, they assessed which structural characteristics (if any) were most important for those playing video games and which features were the most appealing. In devising the list of structural characteristics, they used a number of data sources including (i) reviewing research papers in the field of video game design, (ii) reviewing papers in the field of gambling structural characteristics, (iii) playing a wide variety of video games available at the time of the study, and (iv) interviewing gamers about structural features within video games. Based on these data sources, the main structural characteristics examined in the study were:

- Sound (e.g., in-game sound effects, background music, characters speaking)
- Graphics (e.g., use of realistic graphics, cartoon graphics, full motion video features).
- Background and setting (e.g., video games based on a book, television program, film; use of realistic or fantasy settings).
- Duration of game (i.e., how long video games typically take to finish).
- Rate of play (i.e., how quickly gamers “absorb” or “get into” the video game).
- Advancement rate (i.e., the speed in which the gameplay advances in-game).
- Humor (i.e., the use of humor in-game).
- Control options (e.g., what gamers can control in-game including sound, graphics, and skill settings, choice of control methods).
- Game dynamics (e.g., exploring new domains, completing quests, developing skills, collecting in-game items, surviving in-game, shooting things, solving puzzles, solving time limited problems, building environments).
- Winning and losing features (i.e., losing or gaining points, finding bonuses, re-starting a level, saving regularly).
- Character development (e.g., developing character in-game over time, customizing character).
- Brand assurance (e.g., being loyal to a brand, using celebrity endorsement).
- Multiplayer features (e.g., using multiplayer options, building alliances, beating other players).

Wood et al. [33•] reported that a high degree of realism was important in-game for video game players (i.e., realistic settings, graphics, and sound effects). Other important characteristics included character development, a rapid absorption rate, multiplayer features, and game customization.

Other studies have reported that specific structural characteristic in-game can motivate video game play. For instance, an experiment by Chumbley and Griffiths [34] assessed gamers’ willingness to continue to play a video game based on the level of reinforcement in-game. At a basic level, video games which offered frequent rewards and fewer obstacles provided higher levels of motivation to play among gamers. In a similar study, King and Delfabbro [35] reported that utilization of concurrent in-game reward structures kept gamers playing for longer periods. Wolfson and Case [36] demonstrated that some structural characteristics of video games may affect physiological and cognitive responses while playing. In a small experiment, they manipulated the sound (loud/quiet) and background screen color (red/blue) while participants played a video game. Their results showed that players’ performance on red screens peaked midway through the game and then deteriorated whereas those playing on a blue screen gradually improved during the gaming session. They reported a similar pattern on players’ heart rates and suggested that arousal may be implicated. The study also found that the use of sound on its own had little impact, whereas the combination of red and loud sound was associated with excitement and playing well. A study by Bracken and Skalski [37] described an experiment in which image quality was manipulated. Findings demonstrated that video games with higher quality images led to higher levels of telepresence (i.e., the sense of “being there” [38]) and immersion among players. While these studies are of interest from a structural characteristic perspective, none of them specifically addressed the relationship between such characteristics and problematic/addictive play (although some alluded to it).

## Narrative and Flow in Video Games

Another study that examined a single gaming structural characteristic was by Qin and colleagues [39] who developed a new psychometric instrument to assess story narratives. Compared to more traditional stories (such as those found in books, and radio, television and film drama), video game story narratives are often non-linear and interactive. While books or films simply need to narrate a story, a video game also has to provide a gaming environment through its narrative. The level of game narrative also varies between the video game genres. For instance, most fighting games present little in the way of narrative while other kinds of games such as role-playing games will include much more detailed and intense narratives.

Given that video games are more *interactive* than other media, a video game designer can hide part of the story, encouraging the player to explore the game world. Two main aspects define interactivity. The first is that the story is shaped both by the gamer’s way to interact with the game’s character(s) and by how they choose to overcome the difficulties. The second mainly concerns Massively Multiplayer Online Role-Playing Games (MMORPGS), and relates to the way players interact with each other in-game. The uncertainty (i.e., the player not entirely knowing the narrative and creating it by playing) causes players to move swiftly into the narrative and helps them to more fully understand it. Another important point in the video game narrative is its *game structure* which, according to the conflict-driven model analysis [40], is composed of several conflicts evolving in their level of difficulty.

According to Salen and Zimmerman [41], there are two main structures in understanding video game narrative components: *embedded narrative* and *emergent narrative*. The first is the core narrative that exists in the game without the player intervention. The second is constructed via the player’s interaction with the game. Majewski [42] asserts the interaction between emergent and embedded narrative can be expressed into four main models of narrative structure: (i) a series of pre-set events (i.e., with choices and possibilities between the events), (ii) multiplicity of paths (i.e., each choice leading to a different storyline), (iii) central storyline (i.e., the narrative is fragmented in sub-plots and the player can move freely between those), and (iv) unstructured narrative (i.e., the player creating the whole story by playing).

Chen, Wigand, and Nilan [43] claim that narrative cannot be explored without including the three stages of the flow experience: *antecedents* (i.e., the prerequisites, the skills needed in the activity), *experience* (i.e., factors perceived in the state of flow), and *effects* (the individual’s experience of flow). With respect to the playing of video games, flow refers to the intense enjoyment of immersion while gaming [44]. Consequently, gamers’ sense of time can become distorted without them realizing that they have spent long periods playing the video game [45]. Because this is so rewarding for gamers, some continually engage in the behavior to elicit repeat experiences. Being so engrossed in the game can also provide feelings of escape from the stresses and strains of the real world. Most of the time, this is positive for the gamers but for a small minority, it may be detrimental as they constantly crave repeated emotional “highs” from their video game playing [46].

The study by Qin et al. [39] explored flow and immersion in gaming narratives based on six dimensions: curiosity, concentration, comprehension, control, challenge, and empathy. Firstly, they surveyed 309 participants who completed a 30-item questionnaire and carried out exploratory factor analysis. The questionnaire was then reduced to 27 items and completed by 325 participants for confirmatory factor analysis (almost all participants were ages 20 to 30 years). The confirmatory factor analysis found that a seven-factor model was the best fit (comprising curiosity, comprehension, challenge and skill, empathy, concentration, control, and familiarity) with familiarity being added at the second stage. More specifically, the instrument’s final dimensions based upon the data collected and analyzed were (taken verbatim from the paper, pp. 127–128):

- *Curiosity: Arousal of senses and cognition and attraction to explore game narrative.*
- *Concentration: Ability to concentrate long-term on the game narrative.*
- *Challenge and skills: Some relative difficulty in the game narrative for players and corresponding players’ skills.*
- *Control: Ability to exercise a sense of control over game narrative.*
- *Comprehension: Understanding the structure and content of the storyline.*
- *Empathy: Mentally entering into the imaginary game world while playing the game.*
- *Familiarity: Being familiar with the game story.*

The authors recommend their instrument be used by any researchers exploring the relationship between narrative and immersion, although the applicability in terms of problematic gaming was not mentioned at all in the study.

Hull et al. [46] carried out a small survey study examining whether the structural characteristics of video games, happiness, and flow could predict of video game addiction. Gamers (*n* = 110) were asked questions assessing video game addiction (Game Addiction Scale [47]), the extent to which 24 structural characteristics were integral to video game enjoyment [48•], flow (Flow State Scale [49]), and happiness (Oxford Happiness Questionnaire [50]). Findings demonstrated that decreases in general happiness were the strongest predictor of gaming addiction. In relation to structural characteristics, it was those associated with increased sociability that significantly predicted video game addiction. In total, happiness, the structural characteristics of video games, and flow accounted for 49.2% of the total variance in video game addiction levels. However, it should be noted that one study (a conference paper by Gackenbach [51]) did not find any relationship between the structural characteristics of games and the experience of psychological flow during game play.

Laffan Greaney, Barton, and Kaye [52•] noted that studies examining the links between pleasure, rewards, and video games have yielded inconsistent results (e.g., playing either for immediate rewards such as escapism or for more motivational reasons). They explored the relationship between flow and the structural characteristics within video games. As noted earlier, although the concept of flow in video games can mostly be considered as a positive state, some negative outcomes can rise, such as time loss or anxiety if players spend large amounts of time gaming. More specifically, Laffan et al. investigated the relationships between the structural video game characteristics (e.g., social features, presentation features, punishment features from the taxonomy developed by King et al. [48•]), happiness, and components of video game engagement (e.g., psychological absorption, immersion) via an online survey. The sample comprised an international sample of 207 video game players who answered questions relating to their most played or favorite video game.

The authors reported that flow correlated significantly with the social features, reward features, punishment features, and presentation features although only the punishment features (e.g., restarting a level, losing a life) and presentation features (e.g., graphics, audio effects) significantly predicted flow level after carrying out a hierarchical multiple regression. Flow was most likely to occur when the gamers rated presentation and punishment as important in gameplay. This appears to make sense because the player must encounter some difficulties (via punishment features) and be immersed in the game (via presentation features) to experience flow. General happiness only weakly predicted flow. The authors concluded that presentation and punishment features facilitated flow experiences because the punishment aspects of video game playing may be contributory factors to the difficulty of playing and the amount of effort needed for a state of flow to be reached.

## Structural Characteristic Taxonomies for Video Games

In the gambling studies field, structural characteristic taxonomies have been developed for both online gambling activities [53] and offline gambling activities [6•]. In the video game literature, one of the most comprehensive structural characteristic taxonomy for video games was developed by King et al. [48•]. They utilized and modified known structural characteristics from the gambling studies literature as well as previous research in the gaming literature (most notably that of Wood et al. [33•]) to create their new video game structural characteristic taxonomy. The taxonomy comprised five types of overarching structural features comprising 24 different sub-features that King et al. claimed can influence video game playing behavior. These five types of structural feature are (i) social aspects of gaming (*social features*), (b) gamers’ roles in influencing in-game outcomes (*manipulation and control features*), (c) gamers’ roles in character creation and interactive storytelling (*narrative and identity features*), (d) ways in which gamers win and lose in-game (*reward and punishment features*), and (e) auditory and visual presentation of video games (*presentation features*) (see Table 1). The paper speculated on how these features applied to problematic gaming, but openly acknowledged that which features specifically contributed most to excessive and/or problematic gaming needed further investigation.

**Table 1:** Summary of the five-feature model of video game structural characteristics (adapted and updated from the International Journal of Mental Health and Addiction, Video Game Structural Characteristics: A New Psychological Taxonomy, Volume 8, 2010, page, King, Delfabbro & Griffiths, with permission of Springer)

Feature type	Sub-features	Examples
Social features	- Social utility features	- In-game voice and text chat
	- Social formation/institutional features	- Guilds/clans in MMORPGs
	- Leader board features	- “Hall of fame” high score list
	- Support network features	- Internet forums, strategy guides
		- Some game genres offer international ranking systems (e.g., MOBA, RTS)
Manipulation and control features	- User input features	- “Combos,” “hot keys”
	- Save features	- Checkpoints, “quick-save”
	- Player management features	- Managing multiple resources
	- Non-controllable features	- Scripted events, loading screens
		- Those features are of particular importance in RTS games.
Narrative and identity features	- Avatar creation features	- Choice of sex, race, attributes
	- Storytelling device features	- Cut-scenes, mission briefing
	- Theme and genre features	- “Role-playing,” “shooting”
Reward and punishment features	- General reward type features	-Experience points, bonuses
	- Punishment features	- Losing a life, restarting a level
	- Meta-game reward features	- Xbox 360 achievement points
	- Intermittent reward features	- Increasing difficulty of levels
	- Negative reward features	- Gaining health, repairing items
	- Near miss features	- Difficult “boss” at end of level
	- Event frequency features	- Unlimited replayability of game
	- Event duration features	- MMORPGs have no endpoint
	- Payout interval features	- Rewarded instantly for playing.
		In RTS games, bad decisions can set off a domino effect resulting in a lost game. In MOBA games, losing too many matches will lead to a drop in the ranking of the player. Also, the event duration will vary between the games, MOBA lasting mostly between 20 and 40 min, while RTS might vary importantly (i.e., between a few minutes and an hour).
Presentation features	- Graphics and sound features	- Realistic graphics, fast music
	- Franchise features	- Trademarked names, e.g., Mario
	- Explicit content features	- Violence, drug use, nudity
	- In-game advertising features	- Real-life brands, sponsors logos
		- Despite becoming very popular, MOBA games do not really have franchises yet, with only *Defense of the Ancient* having a sequel.

Consequently, the same team [54•] carried out an empirical study to investigate the role of structural characteristics in gaming among sample of 421 gamers (aged 14 to 57 years). Utilizing an online survey, gamers were asked questions concerning their video game playing behavior, their interaction with video game structural characteristics, and problematic gaming. Findings demonstrated that structural characteristics comprising reward and punishment features (e.g., leveling up, finding rare items, earning points, fast loading times [i.e., high event frequency]) were the most enjoyable and important aspects of gaming. The results also showed that narrative elements (e.g., participation in an interactive story) were highly rated by all gamers, consistent with the idea that interactive video game playing experience is important for its escape-like qualities [55].

Compared to non-problematic gamers, those with a gaming problem reported a significantly higher level of enjoyment of specific structural features including the finding of rare items, watching video game cut-scenes, accessing adult content in-game, and the tactile sensation (i.e., “feel”) of using a game controller. The most highly rated structural features by problem gamers were earning points, managing in-game resources, mastering the video game, and getting 100% in-game. The commonality among these features is that they are those that take up far more playing time than other structural features. Using a multiple regression statistical analysis, it was also demonstrated that specific types of structural characteristic appeared to be better predictors of problematic gaming than factors such as age, gender, and the time spent gaming weekly. The study by Hull et al. [46] outlined above also reported similar results in relation to the structural characteristics most associated with gaming addiction.

Using a different methodological approach, Westwood and Griffiths [56•] developed a structural characteristic taxonomy of video games using Q-methodology (a mixed methods technique that allows researchers to systematically access individual’s “points of view” concerning a particular topic both quantitatively and qualitatively). The authors investigated the psycho-structural elements of video games that most motivated individuals to play. An online Q-sort task was carried out by 40 gamers (38 males) which led to six distinct types of gamers based on factor analysis: (i) *casual* gamers (preference for short games with good graphics or mission-based games; played 8 h a week on average), (ii) *social gamers* (preference for social multiplayer games; played 12 h a week on average), (iii) *solo limited gamers* (no specific game type preference apart from a preference to play on their own; played 13 h a week on average), (iv) *solo control/identity gamers* (preference for story-driven games with character choice or development, particularly role-playing games; played 18 h a week on average), (v) *story-driven solo gamers* (preference for story driven high-definition graphic single-player games; played 17 h a week on average), and (vi) *hardcore online gamers* (preference for online games within wide franchises; played 18 h a week on average). The study also found that the in-game sound effects and graphics facilitated a more immersive and realistic context for the video game’s rewards and storytelling design. Almost all gamers (apart from the solo control/identity gamers) highlighted the necessity for realistic graphics and high-quality sound.

Other taxonomies of video game design have been developed including the “Mechanics, Dynamics and Affect” (MDA [57]) taxonomy and the “Design, Play and Experience” (DPA [58]) taxonomy. The MDA taxonomy describes eight terms (some motivational and some structural) that underpin the features of video games that are fun to players (i.e., *fantasy*, *challenge*, *narrative*, *expression*, *discovery*, *fellowship*, *sensation*, and *submission*). The DPA taxonomy was developed to describe the design for serious video games and added other categories to the MDA taxonomy including *learning*, *competition*, *altruism*, and *physical activity*. Although both of these taxonomies concern video game design, neither features what would classically be seen as featuring structural characteristics and neither has been empirically investigated.

Quick, Atkinson, and Lin [59] extended on previous research into the structural characteristics and design features of video games by including personality variables. They noted these previous studies and taxonomies focused on frequent gamers or on specific games (e.g., serious games) or gamers (e.g., Massively Multiplayer Online Role Playing Game (MMORPG) players), and their study attempted to create a taxonomy working for a wider number of gamers, including 18 different features (*3D graphics*, *realistic graphics*, *collect things*, *search for hidden things*, *explore things*, *fantasy world*, *play with friends*, *single player*, *more than one player*, *other gender*, *other race*, *other species*, *challenging obstacles*, *difficult to master*, *display skills in public*, *high core*, and *play online*). More specifically, they examined the relationship between structural characteristics and personality by assessing 15 sub-traits of the Five Factor Model using 60 items from the International Personality Item Pool-Neuroticism, Extraversion, and Openness (IPIP-NEO [60]).

They surveyed 293 participants (64% female, median age of 21 years). Two sets of analyses were conducted: firstly, a factor analysis to determine the factors concerning game design, and secondly a cluster analysis to explore associations between video game structural characteristics and personality. The factor analyses on game characteristics yielded six factors explaining 58% of the video game preference variance. These were subsequently labeled *fantasy*, referring to the enjoyment of role playing (13% of the variance); *exploration*, referring to the enjoyment of exploring, collecting, and finding hidden items (12%); *fidelity*, referring to the realism of the game (9%); *companionship*, referring to the pleasure of playing with friends or in multiplayer environments (9%); *challenge*, referring to mastery and overcoming obstacles (8%); and *competition*, referring to displaying one’s skills and competing with others (7%).

The cluster analyses used the six factors above and 15 personality traits (i.e., achievement-striving, activity level, altruism, anger, assertiveness, cooperation, dutifulness, emotionality, excitement-seeking, gregariousness, imagination, morality, self-consciousness, self-discipline, and self-efficacy) to create a typology of six sub-groups of player. The optimal solution contained six clusters:

- *Dutiful companions:* Comprising 21% of the participants and playing for an average 1 h per week. Of these, 27% preferred playing alone, 44% with one other, and 27% with more than one other.
- *Extraverted fidelitist companions:* Comprising 19% of the participants and playing for an average 2.4 h per week. Of these, 26% preferred playing alone, 18% with one other, and 53% with more than one other.
- *Imaginative fidelitist explorers:* Comprising 17% of the participants and playing for an average 3.6 h per week. Of these, 29% preferred playing alone, 31% with one other, and 35% with more than one other.
- *Conscientious companions:* Comprising 16% of the participants and playing on average 1.34 h per week. Of these, 19% preferred playing alone, 46% with one other, and 35% with more than one other.
- *Introverted challenge-seeking fidelitists*: Comprising 15% of the participants and playing on average 1.39 h per week. Of these, 47% preferred playing alone, 33% with one other, and 19% with more than one other.
- *Calm challenge-seeking companions:* Comprising 12% of the participants and playing on average for 1.37 h per week. Of these, 16% preferred playing alone, 9% with one other, and 72% with more than one other.

When comparing the six factors with the MDA [57] and DPE [58] models, it shows that several of the factors were present in the other older models, indicating that such factors appear to be salient in the video game playing experience. Compared to King et al.’s [48•] taxonomy, the study by Quick et al. failed to identify manipulation and control factors, although it gave rise to an exploration factor that was not present in King et al.’s theoretical taxonomy. It should also be noted that the previous theoretical taxonomies only included game-related features, whereas this empirical study also included personality factors, thus leading to player groups not comparable with previous studies. However, this study suffers from a number of limitations. The sample was only recruited from a large public university of the USA, contained relatively few participants, comprised predominantly of females, and (based on the average number of hours per week played) included mainly casual and infrequent gamers. It is highly unlikely that the results are generalizable to more hard-core gamers or those experiencing problems with their gaming. Future research should include an examination of whether personality factors have any relationship with particular structural characteristics (as has been suggested by Peever, Johnson, and Gardner [61]). For instance, it could be that extraverts prefer playing social video games whereas introverts may prefer solo standalone video games.

When discussing taxonomies of structural characteristics, it is also worth noting that there are of course different genres of online games such as Massively Multiplayer Online Role Playing Games (MMORPGs), First-Person Shooter (FPS) games, Multiplayer Online Battle Arena (MOBA) games, and Real-Time Strategy (RTS) games. However, these online game genres are not structural characteristics because structural characteristics are the elements and components of the video games and not the games themselves. For instance, in the gambling studies field, genres equivalent to RTS or MOBA games might be games like online poker or blackjack whereas MMORPGS might have more in common with online bingo because of the highly social elements of the games. Games in both the gambling and video game fields comprise different genres but the genres themselves are not structural characteristics and should not be thought of as such. However, while most research examining problematic gaming has tended to focus on MMORPGs, there is a growing body of research that has compared different genres of online games [62–67], research that is important in itself. With regard to the structural characteristics of different online games, while some of the features will be present in most of types of online games (e.g., presence of a chat, possibility to achieve a ranking), other features differ greatly between the game genres. For example, while FPS games and MOBA games feature relatively stable session duration (i.e., around 15- and 30-min session times respectively), RTS feature greater variation in session duration (e.g., in *Starcraft II*, a short game can last less than 10 min, while others can last much longer such as *Age of the Empire 2*).

## Video Game Structural Characteristics and Game Design Ethics

More recently, Klemm and Pieters [68•] examined some of the ethical issues concerning game design and structural characteristics. They noted that much of the current focus on the ethics of gaming concerns the content of games (such as sex and violence) but that little consideration has been given to game mechanics such as the reward systems that video game developers design into the games to make gamers play for longer. Taking the case of MMORPGs, Klemm and Pieters argue that such games can affect players differently, and can lead to addiction via motivations or attraction. Here, the authors argue that “attraction addiction” is where the content and design of the video game facilitates addiction, whereas “motivation addiction” facilitates the development of addiction via the human aspects (including psychological factors such as personality characteristics).

However, as a number of authors have pointed out, structural characteristics and individual characteristics can impact on each other. Griffiths [2, 3] noted with regard to structural characteristics in slot machine gambling that such characteristics can potentially be differentiated as either a “pure” structural characteristic (i.e., what the developer designs into the machine game) or a psycho-structural characteristic (i.e., an individual’s relation or reaction to a particular structural characteristic within a game). The same observation has also been made in relation to the structural characteristics of video games [33•]. Similarly, Hamlen [69] noted that the problem of approaching motivation from either a game structure perspective or a psychological motivation perspective is that video games “must be designed in such a way as to elicit these feelings and responses from the player, thus involving both game design and intrinsic motivation” (p. 533).

Klemm and Pieters also note that when game developers create software for a video game, they will “impose” a behavior to the user either consciously (i.e., coding, knowing what behavior they want to achieve) or unconsciously (i.e., behavior as an unexpected a side effect). They claim that coding to modify a video game player’s behavior is common in MMORPGs (e.g., coding reward systems so that gamers will play for longer). To help overcome problematic play, they assert that game designers should incorporate “technological mediation” (i.e., coding video games to avoid or discourage misuse). The purpose of such mediation is not to force players to adopt a particular behavior but to make them “choose” a more constructive behavior rather than a more “destructive” one. The authors claim that such mediations could work via emotions on three different levels (i.e., visceral, behavioral, and reflective) but focus their analysis on the behavioral level and examine the “function and use of a product” as they believe that this is the most crucial aspect in gaining insight into the development of problematic gaming.

Klemm and Pieters claim that several game mechanics trigger player emotions, and thus impact on their subsequent behavior (mainly resulting in the continuance of playing within session). One game mechanic included in all MMORPGs is the reward system involving the use of operant conditioning that leads to repetitive play because the player cannot predict when the next reward (e.g., leveling up, money, valuable in-game items) will be provided within-game. Another game mechanic used in video games to enhance time spent in-game is the social interactions. MMORPGs are designed to make it very hard—if not impossible—in some games to advance in-game alone. Basically, individuals have to form strategic partnerships and work as a team to advance and that some quests within the game can take hours at a time. One person leaving the game—even if they have been playing together for hours—can lead to failure for the whole group in-game. Players become socially obliged to play even if they do not want to or do not really have the time to play.

Klemm and Pieters evaluated the ethics of video game design mechanics utilizing Tromp et al.’s [70] model of technological mediation. According to this model, a design can influence one’s behavior either strongly or weakly, and implicitly or explicitly, leading to four quadrants: *coercive* (i.e., explicit and strong), *persuasive* (i.e., explicit and weak), *seductive* (i.e., Implicit and weak), and decisive (i.e., implicit and strong). The authors claim that the game mechanics of MMORPGs mechanics are mostly seductive, although some players may be aware of such mechanics, shifting such mechanics to being persuasive (e.g., the probability of getting an item will be hidden from the players). Such mechanics do not have to be negative, as some MMORPGs include game mechanics reducing the time spent in a single session (e.g., logging off for a few hours to gain extra experience points next time the player logs onto the game). However, many games require daily log-ins to the game to gain experience bonuses).

The authors also offer several ways to improve the game mechanics in a way that would lead to less problematic use. For example, more transparent fixed chances to get an item would allow the players to know how long they may need to play before getting an item, thus preventing some from engaging in a long activity. They also propose a linear leveling system where it would take as much time to get between levels 1 and 10, as between 40 and 50. Some countries (e.g., China, South Korea) have introduced “fatigue systems” for minors who after 3 h of gaming, start to punish the individual if they continue the play the game (e.g., getting only half of the experience points needed to advance in the game in the fourth or fifth hour of gaming, and no points after 5 h of gameplay) [71].

Such examples have a purpose to remove game mechanics that may lead to addictive use, but Klemm and Pieters highlight other game mechanics that promote a healthier use of video games. For example, the awarding of badges, rewards, or bonus experience for players not connecting more than 20 h per week. Furthermore, displaying the time spent on the game clearly to players would help them realize when they have been playing for too long, helping them to reduce the length of future sessions. Such recommendations also echo the thoughts of other scholars [72, 73] who have recommended that (like the gambling industry) the video game industry should espouse a duty of care for their clientele and should incorporate policies and strategies that facilitate social responsibility, responsible gaming, player protection, and harm minimization among video game players. It has also been noted that gambling games and video games are beginning to converge both within online video games (e.g., in-game add-ons such as “skins” being used as virtual currency to bet on professional gaming [eSport] tournaments) and via specific online media such as social networking sites (e.g., where gambling-type games like slot machines and poker can be played for points rather than money—so-called social gambling or freemium play where the games can be played for free up to a point but then require payment to get to higher levels or to attain prized in-game assets and items) [74–76]. Whether these new features are structural characteristics is debatable depending upon the definition of structural characteristics used, but these evolving features highlight that the field is ever-moving and that researchers also have to constantly update their research agendas to move with the times.

## Concluding Comments

Compared to other areas such as those examining the psychological and biological underpinnings of problematic and addictive video game play, far less research has examined from a structural characteristic perspective. This is also mirrored in other similar areas (such as the research investigating problematic and addictive gambling) but even when compared to other similar areas, there is still far less empirical evidence in the gaming studies field. Eclectic and multitheoretical approaches are needed if we are to understand the relationship between structural characteristics and video game addiction. Based on the evidence to date, the research appears to demonstrate that some structural characteristics of video games at the very least have a contributory role in the acquisition, development, and maintenance of problematic and addictive video game playing. Clearly more research is needed to establish which structural features appear to be most associated with problematic gaming but it appears that features that take a long time to achieve in-game are (unsurprisingly) the ones rated highly by problem players (e.g., earning experience points, managing in-game resources, mastering the video game, getting 100% in-game).

There is clearly robust theory underpinning why some structural characteristics contribute to addictive video game playing. For instance, operant conditioning helps explain why gamers experiencing problems highly rate structural features such as experience points, rapid loading times, meta-game rewards, and collecting rare in-game items. Many of these rewards operate on variable reinforcement schedules, known to facilitate a repetitive response patterns to stimuli over time and which are often resistant to behavioral extinction. However, there are many structural characteristics where operant conditioning theory is less able to explain the rewards gained by players when interacting with a video game (e.g., game narratives, high-definition graphics, realistic sound features).

Given the recent emergence of research in this particular area, it is unsurprising that many of the studies carried out to date are small-scale, comprise self-selected convenience samples, and are tentative in their conclusions. Most of the research in the area has utilized self-report survey studies or non-ecologically valid laboratory experiments. Future studies should perhaps explore the nature and experience of structural characteristics while gamers are actually playing in their normal gaming context, rather than via retrospective self-report surveys or playing games for unnatural restricted periods in laboratory experiments. One way forward in the field would be for gaming operators to provide access to real-time behavioral tracking data of players so that researchers can conduct secondary analysis of player behavior in relation to specific in-game structural characteristics. This has already started to happen in the gambling studies field with analyses of the impact of structural and situational characteristics on the behavior of slot machine players [77, 78].

The further study of video games from a structural characteristic perspective is of benefit to many different stakeholders. This includes (i) academic researchers who can further understand and integrate a structural perspective into the biopsychosocial model of addiction and apply this knowledge to assist problematic games and help develop effective prevention and intervention programs and strategies, (b) video game players, who can be further educated concerning potentially harmful structural features which may help prevent problematic play, and (c) video game designers, who can then develop video games to include more appealing and rewarding features that promote long-term consumer loyalty without exploiting players. Other stakeholders that may benefit are policymakers and those interested in prevention who could apply some of the findings outlined into their own policies and programs. Video game technologies are ever evolving and becoming increasingly varied and interwoven into contemporary society. Therefore, it is important that researchers understand and recognize the psycho-social effects and impacts that the structural characteristics of video games can have on players, both positive and negative.
